# Assessment of 28-day survival of patients with sepsis based on vitamin D status: a hospital-based prospective cohort study in Indonesia

**DOI:** 10.11604/pamj.2023.45.76.36336

**Published:** 2023-06-02

**Authors:** Rizka Humardewayanti Asdie, Deshinta Putri Mulya, Maria Nainggolan

**Affiliations:** 1 Division of Tropical Medicine, Department of Internal Medicine, Public Health and Nursing, Gadjah Mada University, Yogyakarta, Indonesia,; 2 Division of Allergy Immunology, Department of Internal Medicine, Public Health and Nursing, Gadjah Mada University, Yogyakarta, Indonesia,; 3 Department of Internal Medicine, Faculty of Medicine, Public Health and Nursing, Gadjah Mada University, Yogyakarta, Indonesia

**Keywords:** Sepsis, vitamin D, 28-day survival

## Abstract

**Introduction:**

sepsis is a potentially life-threatening condition caused by the body´s response to an infection. Recent studies have demonstrated a correlation between low vitamin D status and high mortality in septic patients. This study aims to evaluate the vitamin D status of septic patients at Dr. Sardjito Hospital and describe 28-day survival with very low vitamin D levels (< 8.1 ng/mL).

**Methods:**

this prospective cohort study was conducted in the intensive care unit and internal medicine ward at Dr. Sardjito Hospital in septic patients admitted between December 2018 and October 2019. Vitamin D [25(OH)D] was prospectively measured within 24 hours of admission. Data collection used SPSS software for statistical analysis. In addition, the sample size was calculated using the sample size formula used in a comparative survival study intended to find the incidence rate in septic patients. The minimum sample for each group is 23 samples.

**Results:**

sepsis-related mortality was higher in patients with low vitamin D. The analysis included 88 septic patients during the study period. The mean age was 56.09 ± 16.82 years and the proportion of males was 46.6%. 26 of 28 patients with vitamin D levels < 8.1 ng/mL died (92.6%), whereas 39 of 60 patients with vitamin D levels ≥ 8.1 ng/mL (65%) died. Multivariate Cox regression analysis showed that vitamin D concentrations < 8.1 ng/mL at admission (p=0.01) and sepsis shock (p=0.02) were associated with increased sepsis mortality. The hazard ratio of 28-day mortality was 1.95 (95% CI 1.15-3.29, p=0.01) for vitamin D levels < 8.1 ng/mL. The average survival was 9 days for patients with vitamin D levels < 8.1 ng/mL (median: 6 days) compared with 14 days for those with vitamin D levels ≥ 8.1 ng/mL (median: 10 days).

**Conclusion:**

low serum vitamin D levels (< 8.1ng/mL) at admission were associated with increased 28-day mortality in septic patients.

## Introduction

Sepsis is a common condition and a major cause of morbidity and mortality. The documented incidence of sepsis worldwide is 1.8 million cases annually [1]. The case-fatality rate depends on the setting and severity, reaching 30% for sepsis, 50% for severe sepsis, and 80% for septic shock [2]. Vitamin D receptors are expressed in nearly all cells in the body, and the activating enzyme 1-alpha-hydroxylase is expressed in many tissue types. Vitamin D receptors are expressed in immune cells involved in both the innate and adaptive immune responses. A form of vitamin D, 1,25-dihydroxyvitamin D3, appears to serve as a modulator of the immune system [3-5]. There is growing evidence of a close relationship between vitamin D deficiency and various systemic disorders that increase morbidity and mortality in the general population [6].

Vitamin D deficiency was reportedly common in hospitalized patients, particularly critically ill patients. The incidence of vitamin D deficiency in critically ill patients ranges from 26% to 82%. Otherwise, vitamin D deficiency was common in critically ill patients (69%), but it was not an independent risk factor for mortality [7]. It may exacerbate existing immune and metabolic dysfunction in critically ill patients, leading to worse outcomes. A report by Ginde *et al*. found that vitamin D deficiency was associated with increased sepsis severity in emergency department patients hospitalized for suspected infections [8]. The purpose of this study was to investigate the clinical outcomes of septic patients with extremely low 25(OH)D concentrations (< 8.1 ng/mL), to demonstrate the relationship between vitamin D status and mortality, and to describe 28-day survival of septic patients with very low vitamin D levels (<8.1 ng/mL).

## Methods

**Study design and setting:** this study used an observational prospective cohort in the intensive care unit and internal medicine ward. This hospital-based study was conducted during 11 months with patients admitted to Dr. Sardjito Hospital, a tertiary care and teaching hospital in Yogyakarta, Indonesia from December 2018 to October 2019.

**Figure 1:**
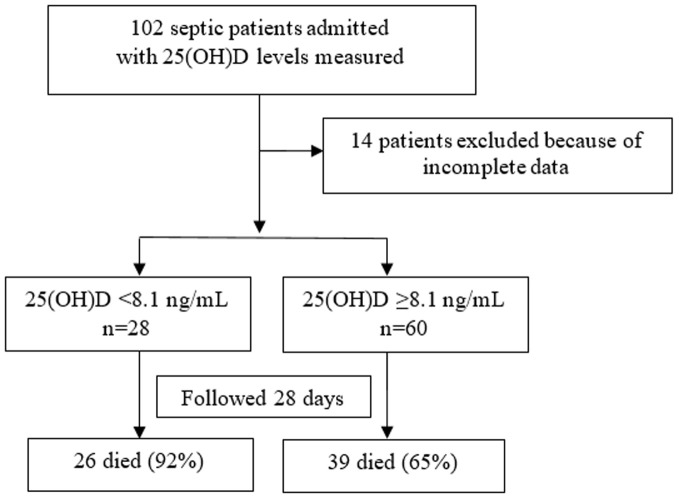
study design

**Study population:** patients with the diagnosis of sepsis were considered for enrollment. The criteria used for the diagnosis of sepsis were adopted from the 2016 Third International Consensus Definition of Sepsis and Septic Shock [9]. The patients were included if they were ≥ 18 years of age and the patients or their guardians agreed to participate and meet the criteria for sepsis. Patients were excluded if microbiological and chemical data were lacking. Levels of 25(OH)D were measured in septic patients within 24 hours of admission. The sample size was calculated using the sample size formula used in a comparative survival study intended to find the incidence rate in septic patients. The minimum sample for each group was 23 samples. All patients received empirical antibiotics upon diagnosis according to the hospital practice guideline. The antibiotics were escalated or de-escalated according to culture reports. Other treatments (standard supportive treatment, fluid resuscitation, vasoactive drugs, medical and technological interventions) were conducted according to “Surviving Sepsis Campaign: International Guidelines for Management of Sepsis and Septic Shock: 2016” [1] (Figure 1).

**Data collection:** baseline demographic data (age, gender, and body mass index), comorbidities, SOFA scores, and primary sources of infection were recorded. Clinical and laboratory variables obtained within 24 hours of hospital admission were complete blood count, serum creatinine, albumin, cultures, and 25(OH)D. All tests were performed by routine methods employed at biochemistry laboratory of Dr. Sardjito Hospital. Patients enrolled in the study were followed until ICU or hospital death or hospital discharge. Three investigators assed the patient for the eligibility for inclusion in the study. All the data were revised by two senior investigators (RHA, DPM), if any problem was detected, patient history was evaluated. All patients were followed up for 28 days since sepsis diagnosed. Surviving patients were censored after 28 days of follow-up.

**Laboratory analysis:** 25(OH) vitamin D is considered the best parameter for the definition of vitamin D status. Serum 25(OH)D concentrations were assessed using the VIDAS 25(OH) Vitamin D Total assay (IFCC-EUROLAB). The assay´s limit of detection for 25(OH)D is 8.1 ng/mL, with a reportable range of 8.1 to 126 ng/mL [10]. Diagnoses of comorbidities and the source of infection were established according to the clinical and laboratory criteria. Blood cultures were processed with the BACTEC system at the Dr. Sardjito Hospital microbiology laboratory according to the Clinical Laboratory Standards Institute (CLSI). The Sequential Organ Failure Assessment (SOFA) was used to assess the severity of organ dysfunction in septic patients using PaO2, platelet count, creatinine level, and bilirubin level data. Albumin was examined by spectrophotometry using the bromocresol green reagent on the Roche Cobas 6000 instrument in the clinical pathology laboratory of Dr. Sardjito Hospital. Dr. Sardjito Hospital, the academic medical center hospital of Gadjah Mada University, has been accredited by Joint Commission International (JCI) since 2014.

**Definition of vitamin D:** according to the Endocrine Society Clinical Practice Guidelines, vitamin D deficiency was defined as a serum 25(OH)D level of 0.0 to 19.9 ng/mL, insufficiency as 20.00 to 29.9 ng/mL, and sufficiency as ≥ 30 ng/mL. The minimum desirable serum level of 25(OH)D is ≥ 30 ng/mL [11]. Studies examining 25(OH)D deficiency in intensive care units have used different cutoff levels for critically ill septic patients. In our study, we used a 25(OH)D cut-off level of < 8.1 ng/mL, which was the lowest threshold of our detection test. Because of the high rate of very low vitamin D levels in critically ill septic patients and its presumed strong association with worse clinical prognosis, a group of patients with very low vitamin D levels (< 8.1 ng/mL), was identified and analyzed as the exposure group.

**Endpoint:** the primary endpoint was clinical outcome defined as death within 28-days following sepsis (28-day mortality). Surviving patients were censored after 28 days of follow-up.

**Statistical analysis:** the Kolmogorov-Smirnov test was used to evaluate the distribution of variables. Continuous variables are expressed as means ±standard deviation (SD). Continuous variables were compared using t-tests for normally distributed data and Mann-Whitney tests for non-normally distributed data. Nominal variables, such as gender and other comorbidities, were analyzed using Chi-squared or Fisher exact tests. Vitamin D status was determined by serum 25(OH)D levels. For statistical comparison, we categorized the patients into two groups: vitamin D levels < 8.1 ng/mL and vitamin D levels ≥ 8.1 ng/mL (according to the lowest threshold of our detection test). SPSS software (SPSS version 22) was used for statistical analysis. A survival comparison between the two groups was accomplished using the Kaplan-Meier estimation. Cox regression analysis was used to determine the hazard ratio. The multivariate Cox regression model retained any covariates associated with 28-day mortality (p < 0.25) in the univariate Cox regression analysis. For all tests, p < 0.05 was considered statistically significant.

**Ethical consideration:** the study was approved by the Medical and Health Research Ethics Committee of the Faculty of Medicine, Public Health, and Nursing at Gadjah Mada University and granted the ethical clearance letter of KE/FK/1049/EC/2019. All subjects or their guardians have received informed consent and agreed to participate in the study as shown by their signing in the informed consent forms.

## Results

**General characteristics of the study population:** a total of the 88 included patients, 41 were men (46.6%) and 47 were women (53.4%), with a mean age at the admission of 56.09 ±16.82 years. The mean SOFA score was 7.26±3.6 and the mean ± SD 25(OH)D concentration was 15.25±9.14 ng/mL. The most frequent infection was pulmonary infection (n= 41, 46.6%). The most frequently used empirical antibiotic was cephalosporin (39.8%). Positive blood cultures were found in 33% of patients. Septic shock was encountered in 56 patients (63.6%), and acute kidney injury was encountered in 45 patients (51.1%). The 28-day mortality rate of the entire study population was 73.9%. There were no significant differences regarding age, BMI, disease severity (according to the Sequential Organ Failure Assessment), albumin, comorbidities, blood culture, and septic shock between the two groups (Table 1).

**Table 1:** demographic and clinical characteristics based on vitamin D levels

Variable		Total study population	Vitamin D <8.1 ng/mL	Vitamin D ≥8.1 ng/mL	p-value
		n= 88	n=28	n =60	
Age, years (mean±SD)		56.09±16.82	51.36±17.63	58.30±16.10	0.71
Gender	Male n (%)	41(46.6)	8 (28)	33 (55)	-
	Female n (%)	47(53.4)	20 (71.4)	27 (45)	-
BMI, kg/m2(mean±SD)		20.92±4.33	20.71±4.8	21.02±4.1	0.77
SOFA score(mean±SD)		7.26±3.6	6.21±3.2	7.75±3.6	0.08
Albumin, mg/dL(mean±SD)		2.56±0.63	2.6±0.69	2.5±0.60	0.30
Comorbidities N (%)	< 2 comorbidities	43(48.9)	12 (42.9)	31(51.7)	0.44
	≥ 2 comorbidities	45(51.1)	16 (57.1)	29 (48.3)	-
Source of infection n(%)	1 source of infection	59(67)	15 (53.6)	44 (73.3)	0.06
	≥ 2 sources of infection	29(33)	13 (46.4)	16 (26.7)	-
Empiric antibiotic n (%)	Penicillin	1(1.1)	0	1 (1.7)	-
	Cephalosporin	35(39.8)	9 (32.1)	26 (43)	-
	Carbapenem	23(26.1)	6 (21.4)	17 (28.3)	-
	Quinolone	1(1)	0	1 (1.7)	-
	Antibiotic combination	28(31.8)	13(46.4)	15 (25)	-
Positive blood culture		29(33)	9 (32.1)	20 (33.3)	0.91
Septic shock n(%)		56(63.6)	17(60.7)	39 (65)	0.69
AKI n (%)		45(51.1)	18(35.7)	25 (58.3)	0.48

**Abbreviations**: BMI, body mass index; SOFA, sequential organ failure assessment; AKI, acute kidney injury.

**Mortality:** only vitamin D levels < 8.1 ng/mL on admission (HR 1.90, 95%CI 1.14-3.15, p=0.01), pulmonary infection (HR 1.93, 95% CI 1.03-3.63, p=0.04), and septic shock (HR 1.75, 95% CI 1.03-2.97, p=0.03) were significantly associated with mortality. Multivariate Cox regression analysis was performed to identify factors independently associated with 28-day mortality in septic patients. Vitamin D levels of < 8.1 ng/mL (HR1.95, 95% CI 1.15-3.29, p = 0.01) and the presence of septic shock (HR1.94, 95% CI 1.09-3.46, p=0.02) were significantly associated with increased with mortality (Table 2).

**Table 2:** Cox regression analysis

Variable		Bivariate		Multivariate	
		HR (CI 95 %)	p-value	HR (CI 95 %)	p-value
Age (years)	≥65	1.19 (0.69–2.05)	0.52		
	<65	Ref			
Gender	Male	1.07 (0.65–1.74)	0.78		
	female	Ref			
25(OH)D (ng/mL)	<8.1	1.90 (1.14–3.15)	0.01*	1.95 (1.15–3.29)	0.01*
	≥8.1	Ref			
Albumin (g/dL)	<3.5	1.94 (0.60–6.19)	0.26		
	≥3.5	Ref			
Comorbidities	Heart failure	0.89 (0.50–1.60)	0.71		
	Diabetes	1.11 (0.63–1.96)	0.70		
	Stroke	0.66 (0.30–1.46)	0.31		
	COPD	1.10 (0.57–2.10)	0.77		
	CKD	0.94 (0.54–1.64)	0.84		
	Cirrhosis	1.51 (0.79–2.90)	0.21	1.87 (0.94–3.71)	0.71
	Malignancy	1.36 (0.83–2.22)	0.21	1.19 (0.73–1.96	0.47
	≥2 comorbidities	1.02 (0.62–1.66)	0.93		
	<2 comorbidities	Ref			
Source of infection	Pulmonary	1.93 (1.03–3.63)	0.04*	1.58 (0.80–3.13)	0.18
	Urinary tract	0.73 (0.43–1.22)	0.23	0.71 (0.40–1.24)	0.23
	Skin soft tissue	0.95 (0.49–1.82)	0.89		
	Intra-abdominal	0.65 (0.23–1.79)	0.40		
	Infection	0.48 (0.06–3.47)	0.46		
	≥2 sources of infection	1.11 (0.66–1.85)	0.67		
	1 source of infection	Ref			
Positive blood culture		1.55 (0.91–2.52)	0.10	1.59 (9.47–2.66)	0.07
Septic shock		1.75 (1.03–2.97)	0.03*	1.94 (1.09–3.46)	0.02*
AKI		0.68 (0.42–1.12)	0.33		

**Abbreviations**: 25(OH)D, serum 25α-hydroxyvitamin D; CKD, chronic kidney disease; SOFA, sequential organ failure assessment; AKI, acute kidney injury. *indicate a significant p-value.

**Figure 2:**
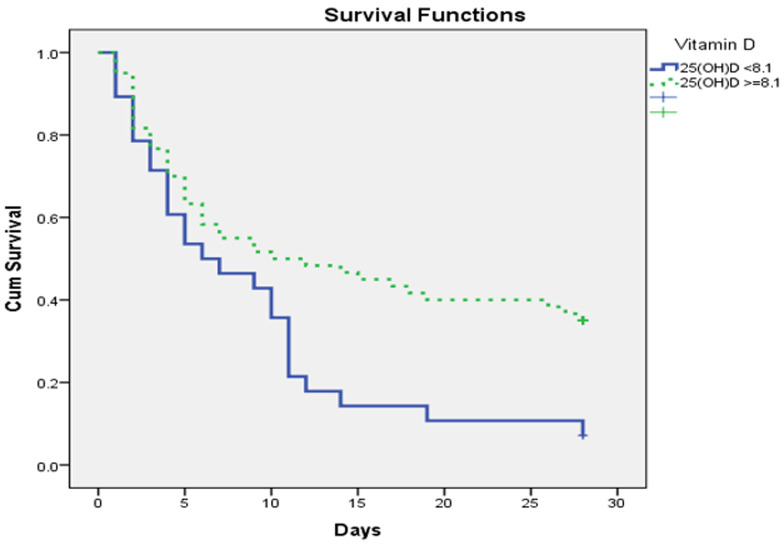
Kaplan-Meier curve for septic patients with 25(OH) D < 8.1 ng/mL

**Twenty-eight-day survival analysis:** for the 28-day observation period, 26 of the 28 patients with vitamin D levels of < 8.1 ng/mL died (92.6%), whereas 39 of the 60 patients with vitamin D levels of ≥ 8.1 ng/mL died (65%). This difference was statistically significant (p=0.006). The overall 28-day survival was 26.1%. The survival rate in patients with vitamin D levels of < 8.1 ng/mL was lower than that in patients with vitamin D levels of ≥ 8.1 ng/mL (7.14% vs. 35%). The more prolonged average survival was noted among patients with vitamin D levels of ≥ 8.1 ng/mL. The mean survival of patients with vitamin D levels of < 8.1 ng/mL was 9 days (median: 6 days), and the mean survival of patients with vitamin D levels of ≥ 8.1 ng/mL was 14.8 days (median: 10 days) (p=0.008) (Figure 2).

## Discussion

This study aims to evaluate the vitamin D status of septic patients at Dr. Sardjito Hospital and describe 28-day survival with very low vitamin D levels (< 8.1 ng/mL). This study used a 25(OH)D cutoff level of 8.1 ng/mL to categorize patients into two groups (vitamin D < 8.1 ng/mL and ≥ 8.1 ng/mL). The results showed that 28 patients (32%) with sepsis had deficient vitamin D levels (< 8.1 ng/mL) and 60 patients (68%) with sepsis had vitamin D levels ≥ 8.1 ng/mL. De Pascale *et al*. reported that 53% of septic patients had undetectable vitamin D levels (vitamin D < 7 ng/mL) [12]. The mean survival of patients with vitamin D levels of < 8.1 ng/mL was 9 days (median: 6 days), and the mean survival of patients with vitamin D levels of ≥ 8.1 ng/mL was 14.8 days (median: 10 days).

This study sought to determine whether vitamin D levels and 28-day survival of septic patients were correlated. In this study, vitamin D levels of < 8.1 ng/mL were associated with a higher mortality rate than those of =8.1 ng/mL (92% vs. 65%). These results are consistent with those of several previous studies, which reported that patients with sepsis who had low vitamin D levels had higher mortality rates compared with patients with sepsis who had higher vitamin D levels [12-14]. This study demonstrated that a vitamin D level of 8.1 ng/mL was the best cutoff to identify patients with sepsis at a higher risk of death. In this prospective cohort study, vitamin D concentrations of < 8.1 ng/mL were associated with higher mortality. Several studies have reported results consistent with our findings, demonstrating similar significant associations [12,13,15-18]. A systematic review and meta-analysis conducted by Upala *et al*. (2015) showed a significant relationship between vitamin D deficiency and sepsis [19]. In another systematic review and meta-analysis, de Haan *et al*. (2014) reported that vitamin D deficiency increased the risk of sepsis and death in critically ill patients [19,20].

Previous research has indicated that vitamin D receptors are expressed in almost all body cells, and its activating enzyme 1-alpha-hydroxylase is expressed in many tissues. Vitamin D regulates the innate and adaptive immune systems; thus, vitamin D deficiency can lead to dysregulation of the immune systems [7,19,20]. The innate and adaptive immune systems are regulated by vitamin D levels, which are related to the production of potent antibacterial peptides such as cathelicidin (LL-37) and ß-defensin-2. In humans, cathelicidin shows potent activity against bacteria (including mycobacteria), viruses, and fungi [21]. In previous studies, cathelicidin levels tended to be low in patients with sepsis and positively correlated with 25(OH)D levels. This finding was confirmed in a cohort study by Leaf *et al*., in which 121 critical patients with low 25(OH)D levels on the first day of treatment in an ICU had low levels of cathelicidin antimicrobial protein-18 (hCAP18), which was also associated with high mortality rates in these patients [22,23].

*In vitro* research on macrophages infected with *Mycobacterium tuberculosis* revealed an elevation in endogenous antimicrobial cathelicidin peptides and an escalation in the killing process of microorganisms. In addition, vitamin D deficiency led to disorders of macrophage maturation, which disrupted their production of surface antigens, lysosomal enzymes, and H2O2, thereby inhibiting their antimicrobial function [24].

This study has several limitations. First, we only assessed the relationship and survival. We did not assess the causal relationship between vitamin D deficiency and mortality. In addition, this research was conducted only at Dr. Sardjito Hospital (a single center) with relatively small sample size. However, this research provides a foundation for conducting larger-scale research or clinical trial research in the future.

## Conclusion

Deficient vitamin D levels, especially 25(OH)D concentrations below 8.1 ng/mL, were associated with higher 28-day mortality in septic patients. A 25(OH)D cutoff level of 8.1 ng/mL predicted mortality in 92.6% of this cohort. The mean survival of patients with vitamin D levels of < 8.1 ng/mL was 9 days (median: 6 days), and the mean survival of patients with vitamin D levels of ≥ 8.1 ng/mL was 14.8 days (median: 10 days) We observed shorter survival among patients with low vitamin D levels (< 8.1 ng/mL).

### What is known about this topic

- *Sepsis is a potentially life-threatening condition caused by the body´s response to an infection;*
- *Vitamin D deficiency is reportedly common in hospitalized patients, particularly critically ill patients;*
- *Vitamin D receptors are expressed in immune cells involved in both the innate and adaptive immune responses*.

### What this study adds

- *Sepsis is a common condition and a significant cause of morbidity and mortality;*
- *The average survival was 9 days for patients with vitamin D levels < 8.1 ng/mL (median: 6 days) compared with 14 days for those with vitamin D levels ≥8.1 ng/mL (median: 10 days);*
- *Vitamin D levels of < 8.1 ng/mL were associated with a higher mortality rate than those of ≥ 8.1 ng/mL (92% vs. 65%)*.
